# Characteristics and outcomes of patients admitted to a tertiary academic hospital in Pretoria with HIV and severe pneumonia: a retrospective cohort study

**DOI:** 10.1186/s12879-022-07522-z

**Published:** 2022-06-15

**Authors:** Veronica Ueckermann, Luricke Janse van Rensburg, Nicolette Pannell, Marthie Ehlers

**Affiliations:** 1 Department of Internal Medicine, University of Pretoria, Pretoria, South Africa; 2 Department of Medical Microbiology, University of Pretoria, Pretoria, South Africa

**Keywords:** HIV, Pneumonia, HAART, TB, ICU, Mortality

## Abstract

**Background:**

Human immunodeficiency virus (HIV) contributes significantly to morbidity and mortality in South Africa. Pneumonia and opportunistic infections remain a major cause for hospital admission among those living with HIV, even in the era of the widespread availability of antiretroviral therapy.

**Methods:**

In this retrospective cohort study, the records of patients admitted with HIV and severe pneumonia, requiring high care/intensive care admission, during a period of 12 months (February 2018 to January 2019) were reviewed. Demographic details, antiretroviral use, HIV viral load, CD4 count, sputum culture results and radiological imaging of patients were recorded. Data was analysed to determine variables associated with mortality.

**Results:**

One hundred and seventeen patient records were reviewed for this study. The patients were young (mean age 38.3 years), had advanced disease with low CD4 counts (mean 120.2 cells/mm^3^) and high HIV viral loads (mean 594,973.7 copies/mL). Only 36.9% (42/117) were on highly active antiretroviral therapy (HAART) on presentation to the hospital. *Mycobacterium tuberculosis* (*M. tuberculosis*) was found to be the cause for pneumonia in 35% (41/117), whilst *Pneumocystis jirovecii* (*P. jirovecii*) was found in 21.4% (25/117). Bacterial pneumonia was the cause in 17.1% (20/117) of patients while no specific aetiology was found in 26.6% (31/117) of patients in the cohort. Mortality among the cohort studied was high (40.1%) and the average length of stay in hospital in excess of two weeks. The need for ICU admission, ventilation and CMV viremia was associated with increased mortality. Chest X-ray findings did not correlate with the aetiology of pneumonia, but multiple B-lines on lung ultrasound correlated with *P. jirovecii* as an aetiology and there was a signal that pleural effusion with fibrin stranding predicts tuberculosis.

**Conclusions:**

Patients studied presented with advanced HIV and were often naïve to antiretroviral therapy. Mortality in this cohort of young patients was high, which emphasis the need for earlier diagnosis and treatment of HIV at a primary care level. Lung ultrasound may have clinical utility in the management of patients with HIV and pneumonia, particularly to diagnose *P. jirovecii* as an aetiology.

## Background

Human immunodeficiency virus (HIV) and the acquired immune deficiency syndrome (AIDS) constitute major public health burdens in Sub-Saharan Africa and in South Africa (SA) approximately 7.7 million people are living with HIV and only around 71% of those are on highly active antiretroviral therapy (HAART) [1]. Furthermore, in the year 2019 approximately 72 000 AIDS-related deaths were recorded in SA [1].

With a significant percentage of the population living with HIV in South-Africa and who are not on HAART, patients often present to the healthcare system with advanced disease, which is associated with poorer in-hospital outcomes [2]. Patients living with HIV in resource-limited countries are more likely to present with severe disease, necessitating intensive care and suffer a higher mortality [3, 4].

In contrast, the worldwide trends for intensive care utilization among patients living with HIV shows a predominance of non-AIDS related causes as the reason for admission, rather than opportunistic infections [4]. These trends reflect the impact of HAART [4]. There is much heterogeneity in the available literature on mortality rates of HIV-positive patients admitted with severe pneumonia, with regards to disease stage, predictors of outcome and HAART use. The available data relating to the disease stage at which a diagnosis is made, as well as the mortality rates of HIV-positive patients in Sub-Saharan Africa shows great variability in practice and outcome among different hospitals [3, 5–12]. Predictors of poor outcome in patients with HIV and pneumonia have not been clearly established.

Balkema and colleagues (2014) studied patients with both HIV and tuberculosis, admitted to an intensive care unit in a resource-limited country. In this study, ICU mortality was associated with AIDS-defining diagnosis, higher APACHE II scores and lower CD4 counts [11]. Another study conducted in a resource limited setting in the United States identified the APACHE II score, multiple organ dysfunction and renal replacement therapy as predictors of mortality [12]. Contrary to the study performed by Balkema and colleagues, this latter study found no correlation between CD4 count or admission diagnosis and prognosis. [13]

In this study, a cohort of patients who were HIV-positive and diagnosed with severe pneumonia, admitted to a tertiary academic referral hospital and requiring either high care or intensive care, were studied to document their clinical characteristics, imaging findings and outcomes.

## Methods

This was a retrospective cohort study, performed at a Tertiary Academic Hospital in Pretoria, South Africa. Ethics approval of the research protocol was obtained from the University of Pretoria Health Sciences Research Ethics committee (ref 267/2017) before commencement of the study and research was conducted in accordance with the Declaration of Helsinki. The records of all adult (> 18 years of age) patients admitted with the diagnosis of both HIV and pneumonia, in the Department of Internal medicine between 1 February 2018 and 31 January 2019 were reviewed. The diagnosis of HIV was coded if there was evidence of two positive fourth generation ELISA tests (Siemens diagnostics, Germany), as documented in the National Health Laboratory Service (NHLS) electronic records. The diagnosis of pneumonia was made by the clinical team who managed the patient.

Respiratory specimens were sent for diagnostic testing as part of standard of care, including: microscopy, culture and antibiotic susceptibility testing, Gene Xpert (Cepheid, South Africa), *Pneumocystis* PCR (Fast Track diagnostics, Siemens, Germany) and in some patients a respiratory viruses panel (Fast Track diagnostics respiratory multiplex assay, Germany). Chest X-rays were performed on admission for all patients, as per the departmental protocol. All lung ultrasounds were performed using a universal probe on the Mindray M7 (Mindray, USA) ultrasound system, by the principal investigator, who was formally trained under Professor D Lightenstein in Paris. The “Blue protocol” [13] was performed at the six points described in the protocol. Patterns described were interstitial pattern, pleural effusion, pneumonthorax or consolidation respectively.

Charts were reviewed to collect the following data: age, gender, co-morbidities, HAART use pre-admission, the level of care (high care or intensive care) as well as the need for life-sustaining therapy such as ventilation, inotropic support and dialysis. The NHLS electronic records were accessed to obtain the: CD4 count, HIV viral load, sputum culture results, cytomegalovirus (CMV) viral load and *P. jirovecii* PCR results. Chest X-rays were accessed on the radiology database, whilst lung ultrasound findings were obtained from the clinical notes. For patients admitted to the intensive care, the simplified acute physiology score (SAPS II) was calculated on the day of admission. Duration of hospital stay was calculated from admission to discharge, and in-hospital mortality documented.

Categorical variables are presented as frequencies with percentages, while continuous variables are presented as means and standard deviation. All statistical analysis was performed on SAS (SAS Institute Inc, Carey, NC, USA) Release 9.4, running under Microsoft Windows for a personal computer. Patient characteristics were compared between groups by using the chi-square test, Fisher’s exact test, analysis of variance, Wilcoxon two-sample test and Kruskal–Wallis test where appropriate. In this study p-values ≤ 0.05 were considered significant. Logistic regression analysis was performed to identify predictive factors for mortality. Ethical approval for the study was obtained before the initiation of the study from the University of Pretoria, Faculty of Health Sciences Research Ethics Committee (Ref 256/2017) and permission from the hospital chief executive officer was obtained to access clinical records.

## Results

Admissions to the department of internal medicine for the period of February 2018 to January 2019 were reviewed. A total of 117 HIV-positive patients presenting with pneumonia were included in the study. All patients were managed in the department of Internal Medicine by teams consisting of specialist physicians, training physicians, interns and medical students.

In the patients studied 63% (73/117) were female and 37% (44/117) were male. The mean age of the study cohort was 38.3 years. In total 58.1% (68/117) patients were not known with a diagnosis of HIV, but it was only made as part of the diagnostic workup during the admission for pneumonia. In terms of the aetiology of pneumonia, pulmonary tuberculosis was diagnosed in 35% (41/117) of the study cohort, while *P. jirovecii* was the associated pathogen in 21.4% (25/117) of patients. The former was diagnosed by TB culture and GeneXpert (Cepheid, USA), whilst the latter by real-time PCR analysis (Fastrack Diagnostics, Siemens, Germany) by the NHLS. Twenty sputum and BAL cultures revealed other bacteria as the aetiology for pneumonia, including: *Pseudomonas aeruginosa* 45% (9/20), *Streptococcus pneumoniae* 30% (6/20)*, Klebsiella pneumonia* 20% (4/20) and *Staphylococcus aureus* 5% (1/20).

Fifty-two percent (61/117) of the study cohort required admission to ICU and the in-hospital mortality was 40.2% (47/117), which was higher than the overall mortality for patients admitted to the department with pneumonia (28.4%). Sixty-one of the patients in the study were admitted to ICU, all were mechanically ventilated, 59% (36/61) required inotropic support and 19.3% (12/61) required renal replacement therapy. The baseline characteristics of the patients studied is shown in Table 1 below.

**Table 1:** Characteristics of patients admitted to high care and intensive care units with HIV and severe pneumonia

Variable	Available data (n)	Number (%)	Mean (std dev)	Max/Min
Gender	117			
F		73 (62.4)		
M		44 (37.6)		
Age (years)	117		38.3 (10.72)	19/65
Newly diagnosed HIV	117	68 (58.1)		
CD 4 count (cells/mm^3^)	114		120.2 (140.7)	0/646
HIV viral load (copies/mL)	78		594,973.7 (1,044,366.0)	0/ 6,755,406
HAART	117	42 (35.9)		
Aetiology of pneumonia	117			
Bacterial		20 (17.1)		
*Mycobacterium tuberculosis*		41 (35.0)		
*Pneumocystis jirovecii*		25 (21.4)		
No culture		31 (26.6)		
CMV viral load > 1 000 copies/mL	116	56 (51.7)		
Length of stay (days)	117		15.8 (16.2)	1/108
SAPS 2	61		61.9 (17.5)	35/98
ICU admission	117	61 (52)		
Ventilation		61 (100)		
Inotropic support		36 (59)		
Renal replacement therapy		12 (19.3)		
In-hospital mortality	117	47 (40.1)		

The data was analysed to determine if any variables predicted mortality. There was no significant difference between survivors and non-survivors in terms of gender (p 0.57), TB culture positivity (p 0.32), *P. jirovecii* positivity (p 0.17) or HIV viral load (p 0.39). A CD4 count of > 200 cells/mm^3^ on admission was associated with better survival (p 0.09). The presence of CMV viremia, with a viral load of > 1000/µL (previously associated with poorer outcomes in the literature) [14], was associated with an increased risk of mortality (p 0.028), as was the need for ICU admission (p < 0.001) and mechanical ventilation (p < 0.001). The results are depicted in Table 2.

**Table 2:** Comparison of survivors and non-survivors

Variable	N	Died	Alive	Crude OR (95% CI)	P value
Gender					
F	73	30	43	1	
M	42	15	27	0.80 (0.36–1.75)	0.57
Age (years)					
19–34	49	16	33	1	
35–49	54	30	24	2.58 (1.16–5.75)	0.02^*^
50–65	14	1	13	0.16 (0.02–1.32)	0.09^*^
HAART on admission					
No	75	32	43	1	
Yes	42	15	27	0.75 (0.34–1.63)	0.46
TB					
No	76	28	48	1	
Yes	41	19	22	1.48 (0.69–3.20)	0.32
PJP					
No	92	34	58	1	
Yes	25	13	12	1.85 (0.76–4.51)	0.18
CMV					
No	60	18	42	1	
Yes	56	28	28	2.33 (1.09–4.99)	0.03^*^
ICU					
No	56	7	49	1	
Yes	61	40	21	13.33 (5.14–34.54)	< 0.001^*^
Mech vent					
No	57	7	50	1	
Yes	60	40	20	14.24 (5.49–37.17)	< 0.001^*^
HIV viral load					
< 1000	13	4	9	1	
> = 1000	63	27	36	1.69 (0.47–6.06)	0.42
CD4					
< = 200	84	39	45	1	
> 200	27	6	21	0.33 (0.12–0.90)	0.03*

^*^Statistically significant

When patients diagnosed with TB were compared to those who did not have TB, there was no significant difference in gender (p 0.87), mortality (p 0.32), ICU admission (p 0.23), CMV co-infection (p 0.15) or HIV viral load (p 0.24). The mean CD4 count of those with TB was lower than the mean for those without (108.8/mm^3^ for those with TB vs 126.3/mm^3^ for those without), but this was not statistically significant (p 0.536).

All patients in the study had imaging in the form of chest radiography, as well as chest ultrasound available. The most common chest X-ray finding, as documented in the clinical notes, was bilateral infiltrates as noted in 68.4% (80/117) of patients. Other patterns described included consolidation in 25.6% (30/117) patients, pleural effusion in 2.6% (3/117) patients and a milliary pattern in one (0.85%) patient. No clear associations between chest X-ray image and aetiology for the pneumonia could be made.

On lung ultrasound, the following patterns were described: consolidation (defined by the “tissue sign” and dynamic air bronchograms), B-profile (≥ 3 B-lines from the pleural line, which is indicative of interstitial infiltrates), effusion, effusion with stranding and pneumothorax (absence of lung sliding and a lung point) [15]. The numbers in each category is illustrated in Table 3 below, and some patients exhibited more than one ultrasound pattern.

**Table 3:** Lung ultrasound findings in patients with HIV and severe pneumonia

Lung ultrasound features (n = 117)	n	%
Consolidation	90	76.9
B-profile	30	25.6
Effusion	15	12.8
Effusion with stranding	3	2.56
Pneumothorax	1	0.85

**Fig. 1:**
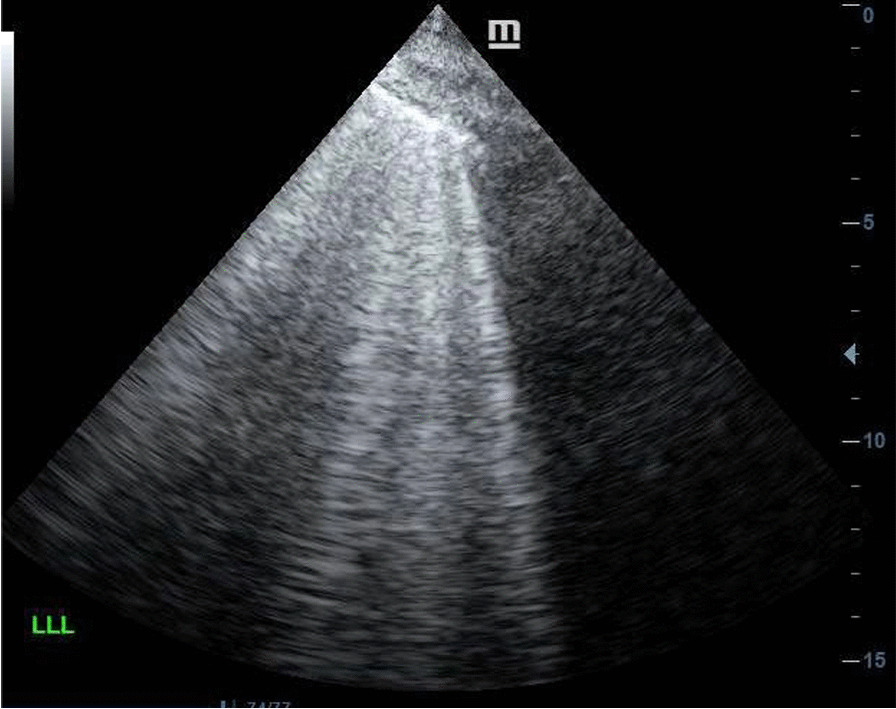
Lung ultrasound image showing the presence of multiple B-lines, the so-called “B-profile” in a patient with *P. jirovecii*

**Fig. 2:**
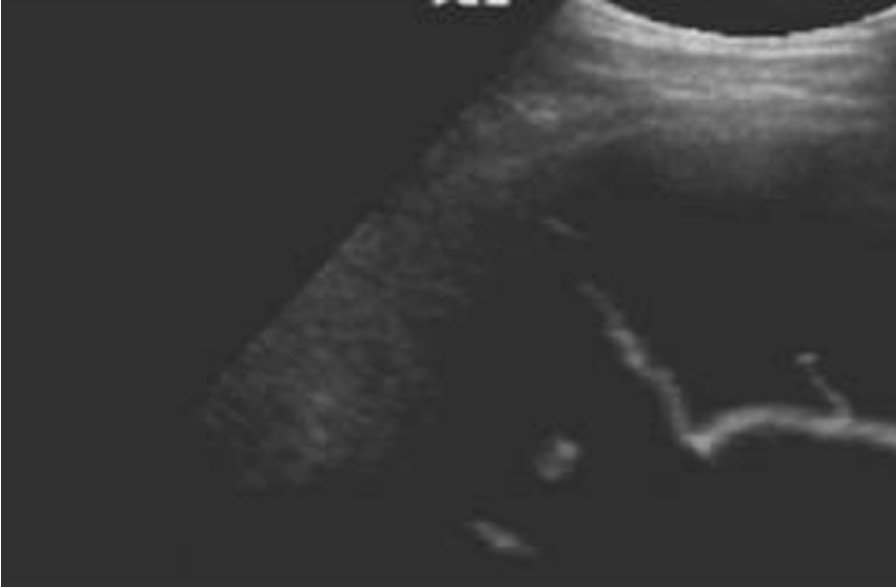
Lung ultrasound image showing a pleural effusion with fibrin stranding in a patient with TB

Figure 1 illustrates an ultrasound image of B-lines, while Fig. 2 illustrates an ultrasound image of pleural effusion with stranding. These images were obtained on the Mindray M7 (Mindray, USA) system, utilizing the universal probe.

**Fig. 3:**
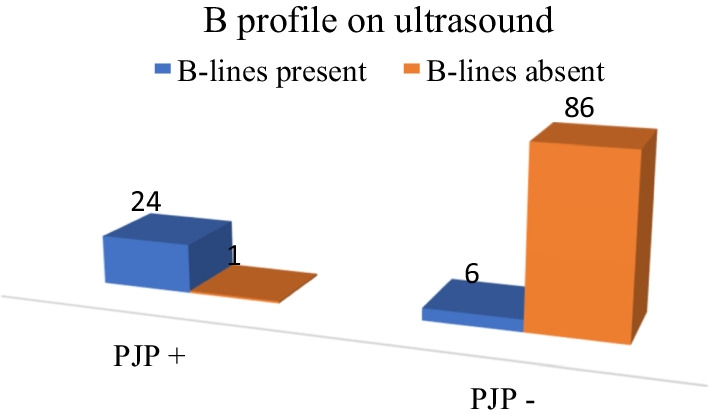
The association between a B-profile on lung ultrasound and *P. jirovecii* as the aetiology for pneumonia in patients with HIV

The presence of consolidation did not correlate with the aetiology of pneumonia, but the presence of more than two B-lines, the so-called “B-profile” on lung ultrasound was associated with a positive *P. jirovecii* PCR test. Twenty-four of the 25 (96%) patients who had *P. jirovecii* exhibited a B-profile and the association between a B-profile and *P. jirovecii* infection was statistically significant (p < 0.001). An additional 5.1% (6/117) of patients had a B-profile, but negative *P. jirovecii* PCR. This is visually represented in Fig. 3 below. The positive predictive value for the presence of B-lines on lung ultrasound in patients with HIV and pneumonia is 80% (95% CI 65.7–94.3).

Three patients in the cohort showed an effusion with evidence of fibrin stranding and all of these were positive for TB on sputum or pleural fluid culture (p 0.017). Pleural effusion was diagnosed on one chest X-ray whilst 15 ultrasound examinations documented its presence. For patients with pneumonia, consolidation was the most common feature. The lung ultrasound findings in each etiological category is summarized in Table 4.

**Table 4:** Lung ultrasound findings according to etiology of pneumonia

Etiology of pneumonia	n	Lung US findings (n)	n (%)
*Pneumocysit jirovecii*	25	B-profile	24 (96)
		Consolidation	9 (12)
		Pneumothorax	1 (4)
*Mycobacterium tuberculosis*	41	Consolidation	30 (73)
		Pleural effusion with stranding	3 (7.3)
		Pleural effusion without stranding	8 (19.5)
Bacterial pneumonia	20	Consolidation	20 (100)
		Pleural effusion without stranding	5 (25)
		B-profile	4 (20)
Unknown etiology	31	Consolidation	31 (100)
		Pleural effusion without stranding	2 (6.5)
		B-profile	2 (6.5)

## Discussion

This retrospective observational study provided some important insights into the characteristics and outcomes of HIV-positive patients admitted with severe pneumonia and highlights the fact that, despite HIV now being a manageable, chronic disease, there is much work to be done in attaining the WHO goal of having 90% of patients diagnosed and 90% of those on appropriate treatment [16]. In this study only 35.9% were on HAART at the time of admission to the hospital and 58.1% were only diagnosed with HIV during the presenting admission. Late diagnosis of HIV has previously been shown to be a risk factor for admission to the ICU [4].

Taking into account the young age (mean 38.3 years) and high mortality (40.1%) in the group studied, it is clear that earlier diagnosis and appropriate treatment is necessary to improve outcomes. The high mortality (40.1%; 47/117) in this study cohort should be contextualised, taking into account that this was a referral centre and only those with severe pneumonia who required admission to high care or ICU. The criteria for admission to high care is the requirement of at least one organ system support (oxygen > 60%, high flow oxygen, non-invasive ventilation, inotropic support or dialysis). Patients managed in the general wards were not included. As more than half of patients (52%) required admission to the intensive care unit, and the mean length of hospitalisation was 15.8 days, it is clear that management is resource-intensive and costly.

Evidence in the literature regarding predictors of outcome in patients with HIV and pneumonia has been heterogenous [4–12] and criteria for admission to critical care units of HIV-positive patients is controversial [4–12]. The CD4 counts and severity-of-illness scores have previously been associated with outcome [11], but in this study no level of CD4 was predictive of mortality, even at counts of < 100 cells/µL. The CD4 of the study population was low with a mean value of 120.2 cells/µL. Furthermore, the need for ICU admission and ventilation increased mortality risk.

In the era of widespread HAART availability, the spectrum of disease seen in people living with HIV and admitted to ICU in developed countries, has shifted to non-AIDS-related illnesses, similar to those without HIV [3]. However, in more resource-limited areas, late-stage diagnosis of HIV with associated opportunistic infections, remains a common reason for ICU admission in developing countries [4, 17]. Chiang and colleagues (2011) did not find the presence of HAART to be associated with better outcomes in patients with HIV admitted to the ICU [10], whilst Morquin and colleagues (2012) found that the initiation of HAART may improve survival in patients with HIV admitted to the ICU [9]. The patients on HAART in this study cohort were more likely to survive than those who were not. In the cohort, 38.6% (27/70) of survivors were on HAART compared to 31.0% (15/47) of non-survivors, but this was not statistically significant (p 0.46). When the subset of 61 patients who required admission to ICU were considered, however, there was a statistically significant survival benefit for those on HAART: 100% (21/21) of survivors were on HAART, while only 25% (10/40) of non-survivors were on HAART (p 0.03). The initiation of HAART in ICU has been associated with improved survival in previous studies [18].

The presence of CMV viremia (viral load > 1000 copies/mL) was associated with a higher mortality (p 0.028). The reactivation of CMV in HIV-positive patients with immune suppression has previously been found to be associated with worse clinical outcomes [19].

Patients with TB admitted to the ICU had a high mortality (33% to 67%) in the published literature [5–13]. A South-African study in patients with TB admitted to the ICU, showed that 53% of participants were co-infected with HIV and the mortality was 59% [11]. In the current study, 35% (41/117) of the patients were found to be culture-positive for TB. There was no statistically significant difference in mortality between those with TB (46.4%) and the cohort as a whole (40.1%).

Imaging by chest X-ray is the first-line imaging for patients presenting with respiratory symptoms. Chest X-ray findings have a wide differential diagnosis, and pneumonia often has overlapping features [20]. The chest X-ray remains a good tool to confirm the diagnosis and look for complications of pneumonia, but it is inferior to computed tomography (CT) in providing diagnostic certainty and establishing a potential causative organism [20]. In this study chest X-ray imaging did not correlate with a specific etiological diagnosis.

Lung ultrasound has become a useful bedside tool in evaluating patients with dyspnea [15] and in those with pneumonia [21]. Although lung ultrasound is comparatively novel when one considers chest X-ray and CT scan as imaging modalities, it has the advantage of being a bedside tool that can be repeated at regular intervals without having to transport patients. In the diagnosis of pneumonia, the pooled sensitivity and specificity were 94% (CI 92% to 96%) and 96% (CI 94% to 97%), respectively. In a small case series Limonta and colleagues (2019) found that all patients with *P. jirovecii* infection had multiple B-lines on ultrasound of the lung; the so-called B-pattern or interstitial pattern [22]. Another, series of 14 patients found B-lines to be 100% sensitive for the presence of *P. jirovecii* and also found subpleural consolidation and cystic changes in patients with *P. jirovecii*. [23] In the current study the presence of B-lines on lung ultrasound were strongly associated with *P. jirovecii* as an aetiology for pneumonia in patients with HIV (positive predictive value 80%, 95% CI 65.7–94.3). Notably the use of lung ultrasound picked up a small pneumothorax, which was not visible on conventional chest radiography.

Kahn and colleagues (2020) considered a combination of ultrasound features to predict TB in patients with HIV and pneumonia. The presence of a pleural effusion argued strongly for TB as the aetiology of pneumonia in this cohort of patients [24]. In this study, 15/117 patients had pleural effusions on lung ultrasound. |Seventy-three percent (11/15) of these proved to have TB as the aetiology for their pneumonia. Three patients were found to have pleural effusion with visible fibrin strands and all three also tested positive on TB culture. This number was too small to make inferences on, but the signal warrants further study in a larger cohort. Larger studies in the future are likely to delineate the role of ultrasound in the diagnosis of TB in the context of HIV to a greater extent. The current study was limited by the small number of patients studied. There was selection bias as in that the study was performed at a referral centre, which means that patients are generally more severely ill.

## Conclusions

In this single-centre retrospective study, the mortality of patients with HIV and severe pneumonia was high (40.1%). Patients admitted were young and the majority were not yet on HAART upon presentation and were newly-diagnosed with HIV during the current admission. The need for ICU admission, ventilation and CMV viral loads of greater than 1000 copies/µL were associated with worse outcomes.

The presence of multiple B-lines on lung ultrasound was useful in predicting *P. jirovecii* as the aetiology for pneumonia in this cohort of patients showing that lung ultrasound may be a useful tool in predicting the causative organism in patients with HIV and pneumonia. Future studies are required to explore the sensitivity and specificity of bedside ultrasound in this context.

## Data Availability

Study data can source documents can be found on the University of Pretoria Data Repository and can be accessed at https://figshare.com/s/4517a87e1a553dffa4f7 and if published the direct link: https://doi.org/10.25403/UPresearchdata.17304131.

## Abbreviations

AIDS: Acquired immune deficiency syndrome
CD4: Cluster of differentiation 4
CMV: Cytomegalovirus
CXR: Chest X-ray
HAART: Highly active antiretroviral therapy
HIV: Human immunodeficiency virus
*P.**jirovecii*: *Pneumocystis* *jirovecii*
SAPS 2: Simplified acute physiology score 2
TB: Tuberculosis
US: Ultrasound
WHO: World Health Organization
